# Physical health monitoring of patients prescribed depot antipsychotic medication in north west Edinburgh community mental health team

**DOI:** 10.1192/bjo.2021.882

**Published:** 2021-06-18

**Authors:** Douglas Murdie, Jakub Wojtowicz, Alexandra Thompson, Anne MacLeod, Adam Mallis, Hamsi Evans, Joshua Haggart, Hae Choi, Vikki Argent

**Affiliations:** 1 Consultant Psychiatrist, NW CMHT, NHS Lothian; 2 University of Edinburgh Medical School - 1st year Medical Student; 3 Consultant Perinatal Psychiatrist, Perinatal MHT, NHS Lothian

## Abstract

**Aims:**

To monitor the quality of physical health monitoring of patients prescribed depot antipsychotic medication in the North West Edinburgh Community Mental Health Team (CMHT). We also evaluated the completeness of prescriptions and Mental Health Act (Scotland) (Act) 2003 paperwork where relevant.

**Background:**

Antipsychotic medications are medicines for treating conditions such as Schizophrenia, but some may be associated with an increased risk of Metabolic Syndrome. Moreover, evidence indicates that patients with major mental disorder have a reduced life expectancy in comparison to those without such diagnoses. These two factors illustrate the importance of the physical health of this patient cohort being monitored on a regular basis. This project will evaluate how a local CMHT is performing, with the possibility of enacting service improvements if required.

**Method:**

The records of the 60 patients prescribed depot antipsychotic medication administered by this CMHT were reviewed. A check-list was created consisting of 14 categories analysing the quality of physical health monitoring, as well as compliance with prescription standards and, where relevant, Mental Health Act (Scotland) (Act) 2003 paperwork. We compared patient records against our checklist for the calendar year of 2019. The Scottish Intercollegiate Guidelines Network (SIGN) 131 (Management of Schizophrenia) section 5.2 was used as the gold standard for physical health monitoring against which the data we collected was compared.

**Result:**

We identified a wide range of flaws with the current system and implementation of monitoring, and difficulty in locating the required information. There was no consistent monitoring of physical observations on electronic record, nor an accepted alternative way in which this was documented. Furthermore, blood tests were not consistently obtained either by the service or GP practices in a reproducible manner. This led to discussions within the CMHT regarding creation of a new pathway for the monitoring of this patient cohort using a Quality Improvement model, with the ultimate goal to establish a regular physical health clinic.

**Conclusion:**

There is significant evidence that patients with major mental disorder do not access healthcare as consistently as those without, leading to a disparity in life expectancy. In light of the fact that antipsychotic medications can be associated with Metabolic Syndrome, we have an even greater responsibility to tackle this marked health inequality by appropriately monitoring our patients. This was not done well in this particular CMHT, but this project will lead to improvements in the service and ultimately patient care.

